# A new BiofilmChip device for testing biofilm formation and antibiotic susceptibility

**DOI:** 10.1038/s41522-021-00236-1

**Published:** 2021-08-03

**Authors:** Núria Blanco-Cabra, Maria José López-Martínez, Betsy Verónica Arévalo-Jaimes, María Teresa Martin-Gómez, Josep Samitier, Eduard Torrents

**Affiliations:** 1 Bacterial Infections and Antimicrobial Therapies Group, Institute for Bioengineering of Catalonia (IBEC), The Barcelona Institute of Science and Technology (BIST), Barcelona, Spain; 2 Nanobioengineering Group, Institute for Bioengineering of Catalonia (IBEC), The Barcelona Institute of Science and Technology (BIST), Barcelona, Spain; 3 Networking Biomedical Research Center in Bioengineering, Biomaterials and Nanomedicine (CIBER-BBN) Monforte de Lemos 3-5, Madrid, Spain; 4 Department of Electronics and Biomedical Engineering, University of Barcelona, Barcelona, Spain; 5 Microbiology Department, Hospital Universitari Vall d’Hebron, Barcelona, Spain; 6 Microbiology Section, Department of Genetics, Microbiology and Statistics, Faculty of Biology, University of Barcelona, Barcelona, Spain

**Keywords:** Infectious-disease diagnostics, Biofilms, Health care, Biological techniques, Clinical microbiology

## Abstract

Currently, three major circumstances threaten the management of bacterial infections: increasing antimicrobial resistance, expansion of chronic biofilm-associated infections, and lack of an appropriate approach to treat them. To date, the development of accelerated drug susceptibility testing of biofilms and of new antibiofouling systems has not been achieved despite the availability of different methodologies. There is a need for easy-to-use methods of testing the antibiotic susceptibility of bacteria that form biofilms and for screening new possible antibiofilm strategies. Herein, we present a microfluidic platform with an integrated interdigitated sensor (BiofilmChip). This new device allows an irreversible and homogeneous attachment of bacterial cells of clinical origin, even directly from clinical specimens, and the biofilms grown can be monitored by confocal microscopy or electrical impedance spectroscopy. The device proved to be suitable to study polymicrobial communities, as well as to measure the effect of antimicrobials on biofilms without introducing disturbances due to manipulation, thus better mimicking real-life clinical situations. Our results demonstrate that BiofilmChip is a straightforward tool for antimicrobial biofilm susceptibility testing that could be easily implemented in routine clinical laboratories.

## Introduction

Biofilms are communities of microorganisms that form on and attach to living and nonliving surfaces. These communities are ubiquitous, as they are found in natural, industrial and medical environments. Biofilms can be beneficial under some conditions, for example, for biodegradation in wastewater treatment, but they are often undesired because of their ability to cause infections, contamination, biofouling, and biocorrosion^1^.

The attached microorganisms form microcolonies on a surface, where the bacteria are embedded in the extracellular polymeric substances (EPS) that form the biofilm matrix^2^. The matrix protects the microorganisms and makes biofilms very difficult to eradicate because it increases their resistance to biological, mechanical, physical, and chemical treatments^3^. Taking into account that approximately 80% of chronic infections in animals and humans are estimated to be biofilm-related^4^, their formation presents a severe threat in the battle against antimicrobial recalcitrance, and related treatments require several billions of US dollars each year worldwide^5,6^. Moreover, to date, no antibiotic that can successfully eradicate biofilms has been found, so there is a great need for new strategies to combat biofilms^7^ while awaiting for the development of effective antibiofilm molecules.

Model systems of in vitro biofilms are essential in research laboratories for testing new antibiofilm compounds, as well as in clinical laboratories for determining the optimal treatment of biofilm-related infections. There is a wide selection of different monitoring techniques for biofilm growth and characterization, varying in the analysis scale, handling time, sensitivity and final detection technique employed^8^. Standard methods mostly rely on colorimetric measurements and are commercially available, i.e., the microtiter plate method^9,10^, the MBEC Assay®^11,12^, the Biofilm Ring Test^13,14^ and the Lubbock system^15^. These techniques allow the screening of different antimicrobials in a high-throughput way, but they are generally destructive endpoint diagnostic tools and require removal of the formed biofilm from the growth substrate used. For this reason, these systems cannot be exploited for online monitoring characterization.

Moreover, the majority of these high-throughput screening techniques involve the use of static devices with limited nutrients, which form biofilms that do not resemble all the biofilms’ characteristics found during natural infections. On the other hand, dynamic devices allow bacteria to grow under flow conditions that eliminate the planktonic growth^16^. Examples of these devices are the Robbins device^17^, the Drip-Flow reactor^18^ or the Rotary biofilm reactor^19^. Although having some limitations (e.g., the prior knowledge of the device flow dynamics, the heterogeneity of the biofilm development, or the low-throughput of these dynamic devices), they can better mimic real in vivo infective conditions^20^. One of the recurrent problems with such flow conditions is the need for large volumes of media and tubing, which prevent high-throughput screening.

Furthermore, most of the available techniques require advanced microscopy systems to monitor biofilm growth^21–24^. Recently, Acea Bioscience (San Diego, USA) released xCELLigence based on electrical impedance spectroscopy (EIS) measured on the basis of a defined Cell Index (CI) parameter^25^. This technique is based on the detection of changes in the diffusion coefficient of a redox solute, which is recorded as an electrochemical reaction on the electrode; thus, this technique provides an excellent nondestructive method for real-time and in situ measurements independent of confocal microscopy. Indeed, EIS was shown to be suitable for online monitoring of biofilm formation, although all the experiments were performed under static conditions and did not resemble all features of natural infections^26,27^.

Microfluidics represents the next generation of fluidic platforms for biomedical research^28^. The Bioflux^29^ and other microfluidic devices^30,31^ provide dynamic systems with significant control over the flow rate settings, potential for real-time analysis and, particularly for biofilms, greater similarity to the in vivo infective environments by creating the physical conditions encountered in natural environments^32^.

Even though antibiotics that affect planktonic growth typically become useless when the bacteria form a biofilm, none of the abovementioned techniques have been used in clinical laboratories to find appropriate treatments for a chronic biofilm infection, as they are too complex and require costly and intricate equipment operated by experienced personnel. Instead, antibiotic efficacy and susceptibilities are determined using planktonic bacteria. Furthermore, real biofilm-forming infections are known to be polymicrobial, which modify their antibiotic susceptibility^33^, but conventional antimicrobial susceptibility test is performed on single isolates obtained from complex samples, thereby missing other important species and their interactions in clinical samples^34^.

In this work, we developed a microfluidic system to grow and analyze biofilms using samples from different sources (in vitro bacteria cultures, clinical samples, etc.) to be used in clinical or industrial settings. This device can be used to determine a personalized treatment for a patient suffering a chronic infection, even though more studies are necessary to validate this. We proposed an innovative rapid method for studying new antibiofouling strategies, including drug susceptibility testing of different bacterial species using EIS. This microfluidic platform allows homogeneous biofilm growth that can be easily monitored without using a confocal microscope and enables the development and co-culture of polymicrobial biofilms that resemble the real biofilm infections found in complex matrixes such as sputum samples (e.g., cystic fibrosis infections).

## Results

### Chip fabrication and characterization

**Fig. 1:**
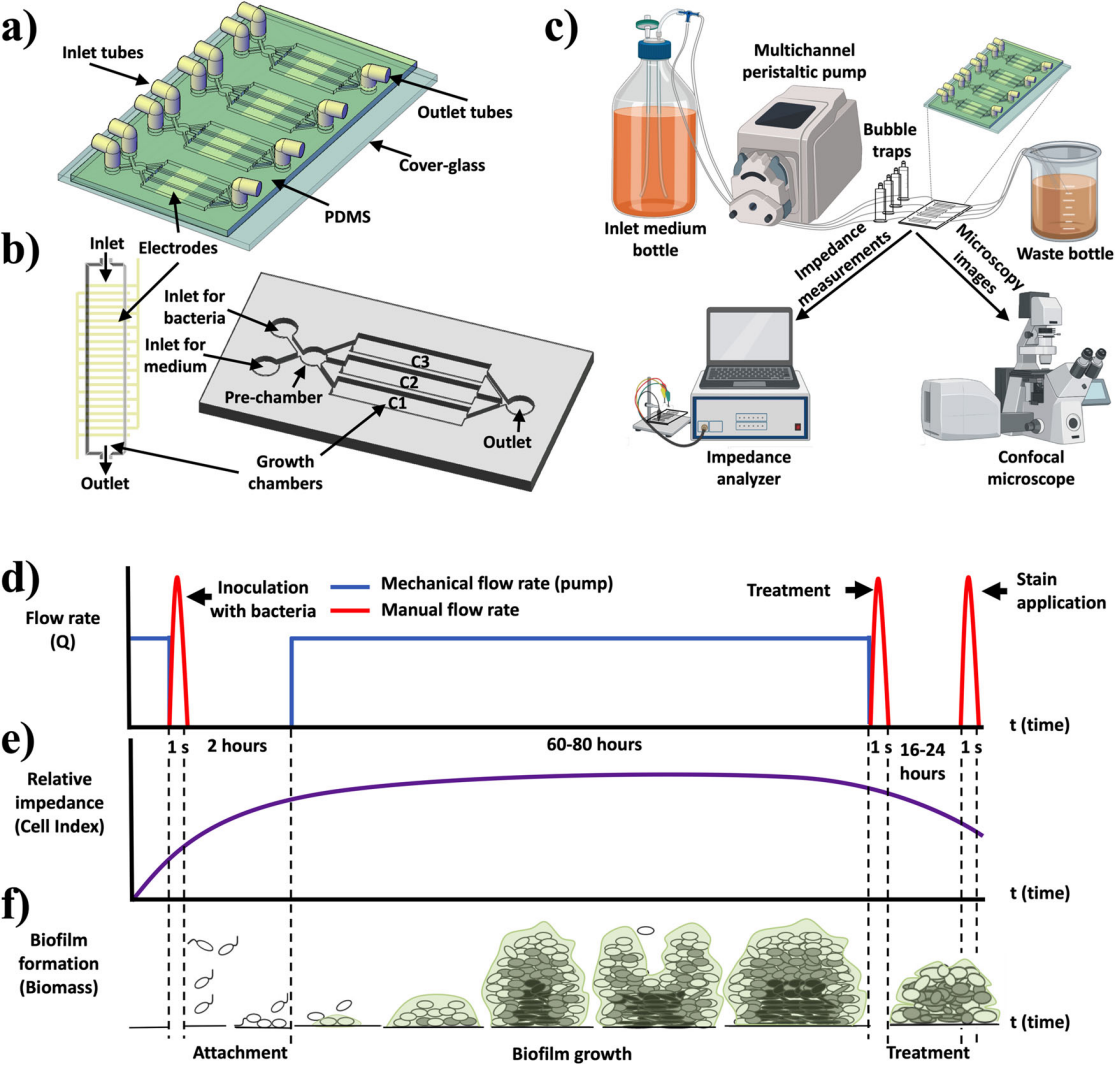
Biofilm chip design. **a** BiofilmChip 3D view, **b** 3D view of one chamber with the electrodes and one set of 3 chambers, **c** experimental setup, **d** changes in the mechanical flow rate (in blue) and the manual flow rate (in red, only during the inoculation with bacteria and applications of treatment or stain), **e** expected relative impedance and **f** biofilm formation over time. Figure created in part with www.biorender.com.

A microfluidic platform with an integrated interdigitated sensor (BiofilmChip) was designed to monitor the growth and treatment of a biofilm in a controlled manner (Fig. 1a, b). The chip was prepared by a combination of standard photolithography and soft lithography techniques. The final system setup is shown in Fig. 1c. Briefly, the medium bottle was connected through tubing to the microfluidic device. Medium was pumped through the system via a high-precision peristaltic pump (see the Material and Methods for details).

Following the experimental scheme shown in Fig. 1d, f, a biofilm formed and grew inside the chambers via irreversible attachment of bacterial cells to the cover glass. The biofilms could then be visualized under a confocal microscope after a specific staining procedure (Fig. 1c, f) or directly analyzed by impedance measurements (as shown in Fig. 1c, e).

### Optimal parameters for uniform biofilm formation

**Fig. 2:**
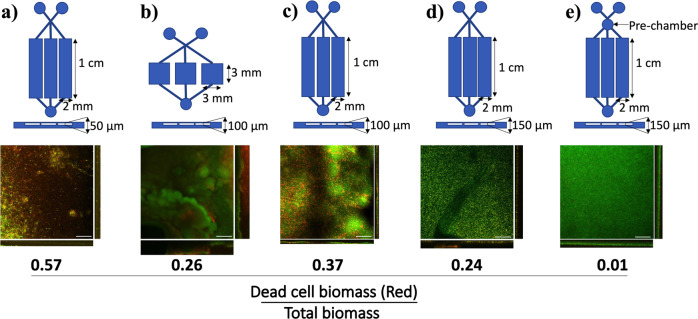
BiofilmChip dimensions, geometry and functional characterization. schematic representations of the different manufactured chip geometries. The *P. aeruginosa* PAO1 biofilm structure formed in each chip is shown in the confocal microscopy images (sum and orthogonal views). In all cases, the biofilm was grown for 72 h and stained with the LIVE/DEAD BacLight Bacterial Viability Kit. At the bottom of the images, average proportion of dead cells (stained in red) in the total biofilm biomass from three different experiments is shown. Scale bar represents 50 μm.

Our primary goal was to design a BiofilmChip with optimized capacity for growing biofilms with maximal uniformity across the different chambers of the chip. First, several prototype designs with chambers that differed in dimension and shape were fabricated and tested by growing a *P. aeruginosa* PAO1 biofilm. The different prototypes manufactured and tested are shown in Fig. 2, and the formed biofilms were established as described in Fig. 1c, stained with LIVE/DEAD dyes, and visualized under a confocal microscope. As seen in Fig. 2, the chamber geometry and height clearly impacted the biofilm formation: a rectangular chamber morphology was preferred over a square morphology. As observed in prototype b (Fig. 2b), the biofilm formed in the square chamber was nonhomogeneous, while that formed in the rectangular chamber (with a height of 150 μm) remained smooth (Fig. 2d).

*P. aeruginosa* cell division was impaired in the outer biofilm layers of the 50- and 100-μm height chambers, with evident cell filamentation and increased cell death (stained in red), indicated by a more significant proportion of red biomass (ratios of dead cell biomass to total biomass of 0.57, 0.26 and 0.37) (Fig. 2a–c). On the other hand, bacteria had a morphology characteristic of wild-type *Pseudomonas* in the chip with 150-μm height chambers and showed uniform green staining covering the entire surface area (a lower proportion of dead cells) (Fig. 2d, e).

Even though the optimal parameters (rectangular shape with 150 μm high) resulted in homogeneous biofilms with a low proportion of dead cells (ratio of 0.24), manual system manipulations (i.e., direct injection during inoculation and staining) caused disturbances in biofilm uniformity (Fig. 2d). There is a tremendous boost of the shear stress all along with the chamber due to the abrupt increase in the flow rate when injecting the bacteria for inoculation. (Fig. 1d). The pressure generated at the inlet was 363 ± 197 psi during manual injection^35^. At this specific pressure generated during injection, we simulated the influence of fluid velocity and chamber shape on biofilm formation using COMSOL Multiphysics software (COMSOL AB, Sweden). Supplementary Fig. 1 shows that the inclusion of a prechamber ahead of the biofilm growth chambers stabilized the flow and rendered a better distribution of velocity, minimizing the effect of sample loading. The results were confirmed by observing uniform biofilm growth in the chambers, as shown in Fig. 2e. Therefore, the prototype with a rectangular chamber, 150 μm high and with a prechamber (Fig. 2e) was chosen as the optimal design for biofilm formation and used thenceforward.

### BiofilmChip robustness

**Fig. 3:**
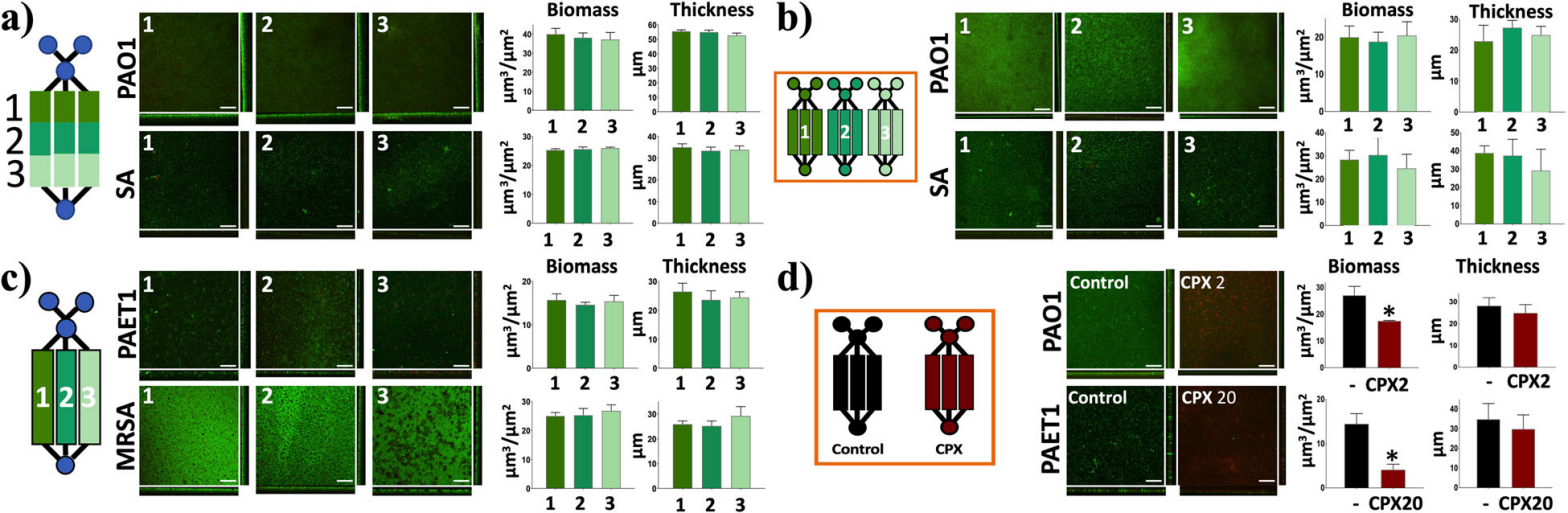
BiofilmChip robustness evaluation. Uniformity in the established biofilm biomass and thickness across different chamber locations (**a**), between sets of chambers (**b**), and among chambers in the same set (**c**). Decreases in biomass and thickness after 24 h of treatment of *P. aeruginosa* PAO1 and PAET1 with ciprofloxacin (**d**). In all cases, 72-hour-old biofilms were stained and visualized by confocal microscopy (CLSM). Images represent the sum of stacked CLSM images and their corresponding orthogonal views, and bars show the quantified biofilm biomass (μm^3^/μm^2^) and average thickness (μm) with error bars of the three different replicates. Scale bar represents 50 μm. The results presented in this figure are representative of the same experiment repeated at least three times, producing similar results every time. PAO1 (*P. aeruginosa* PAO1 laboratory strain), PAET1 (*P. aeruginosa* PAET1 clinical isolate strain), SA (*S. aureus* laboratory strain), MRSA (*S. aureus* MRSA clinical isolate strain), CPX2 (ciprofloxacin 2 µg/ml), CPX20 (ciprofloxacin 20 µg/ml). *p < 0.05 vs. control in a *t*-test.

We next evaluated the robustness of the BiofilmChip with the optimized conditions (rectangular chambers 2 mm wide, 10 mm long, and 150 μm high with a 2-mm prechamber diameter) (Fig. 2e) by analyzing some biofilm parameters along with the biofilm growth chamber. For these experiments, we used laboratory strains of *Pseudomonas aeruginosa* PAO1 and *Staphylococcus aureus* (ATCC12600). We compared the biofilm biomass and thickness at different locations inside a chamber (close to the inlets, the middle part and the outlet, see Fig. 3a), between sets of chambers (Fig. 3b) and finally among separate chambers of the same set (Fig. 3c). In all cases, we observed robust, homogenous biofilm formation with similar biomass and thickness values among the different locations and chambers, demonstrating the uniformity of the biofilm formed. Note that in Fig. 3c, we used two bacterial strains isolated from chronically infected cystic fibrosis patients (*P. aeruginosa* PAET1 and methicillin-resistant *S. aureus* MRSA).

As a proof of concept to evaluate the ability of our BiofilmChip to assess antimicrobial therapy, we determined whether a reduction in the biofilm biomass can be detected with our BiofilmChip, by treating a mature biofilm (72 h old) with the extensively used antibiotic ciprofloxacin (CPX). As shown in Fig. 3d, the treatment of *P. aeruginosa* laboratory strain PAO1 and clinical *P. aeruginosa* isolate PAET1 with 2 and 20 μg/ml ciprofloxacin (10× approximately their MIC, See Supplementary Table 1), respectively, reduced the biofilm biomass (35 and 71%) and thickness (12 and 14%) after 24 h, detected by measuring their viability with the LIVE/DEAD staining. Moreover, this reduction of biofilm biomass correlates with an increase of dead cells caused by the ciprofloxacin (see results in Supplementary Table 2). Therefore, BiofilmChip was suitable for use in inhibition experiments to observe changes in biofilm biomass after specific antibiotic treatment.

### BiofilmChip is a versatile system for growing biofilms directly from clinical sputum samples

The BiofilmChip prototype has been tested with laboratory and clinical strains of *P. aeruginosa* and *S. aureus* (Fig. 3) and directly from patient samples.

Sputum samples from cystic fibrosis patients (Supplementary Table 3) at different stages of *P. aeruginosa* and/or *S. aureus* chronic infection were tested. First, the sputum samples were treated with hypotonic media as described in the Material and Methods section and inoculated directly into the BiofilmChip by injecting the sample through the inlet port while the medium flow was stopped. After 2 h, the TSB media was allowed to flow for 72 h. The formed biofilm was stained and visualized under a confocal microscope.

**Fig. 4:**
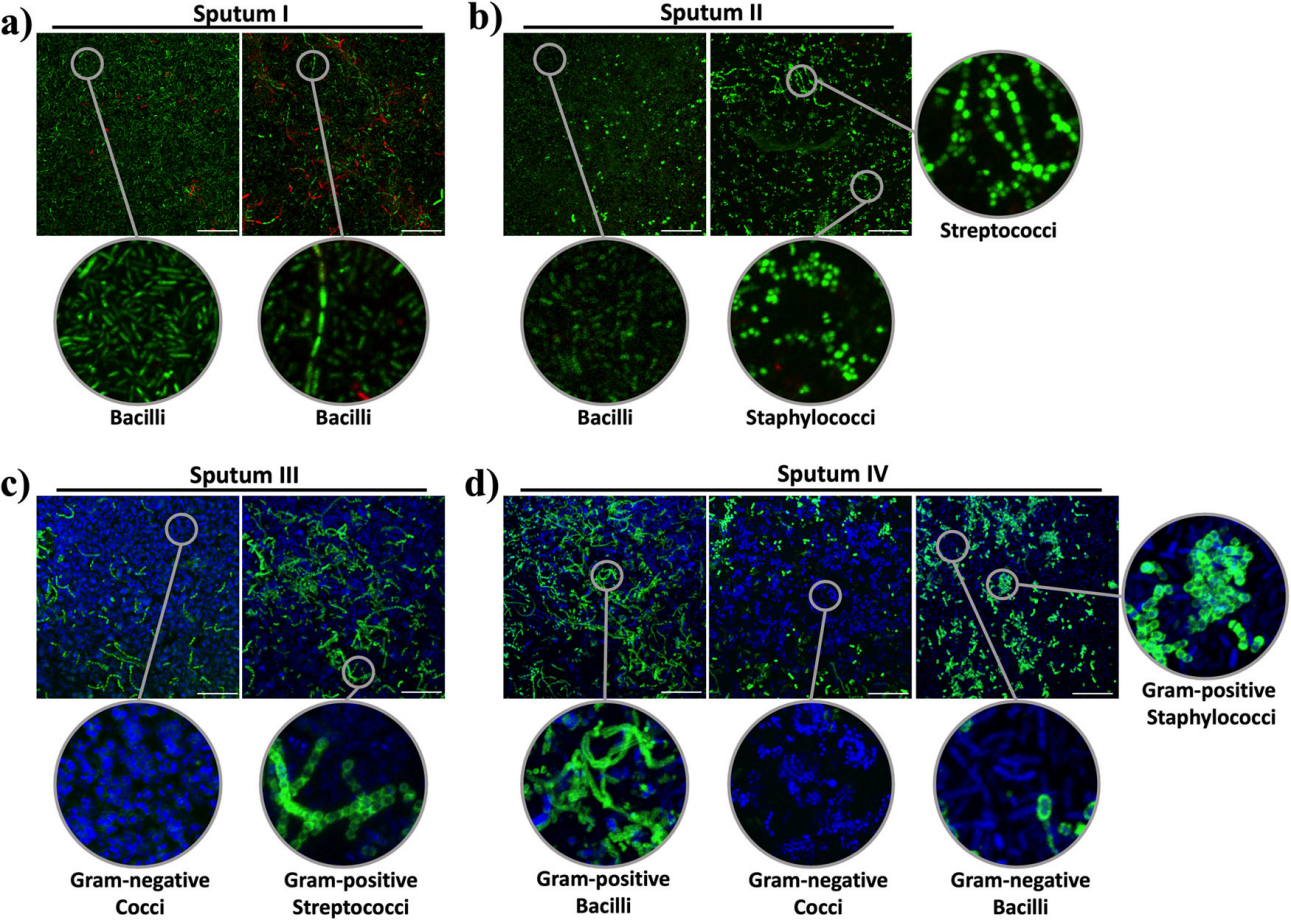
Biofilms formed from four different sputum samples (I–IV) with enlarged images showing the different bacterial shapes found. Biofilms were stained with a LIVE/DEAD BacLight Bacterial Viability Kit (**a** and **b**) and with a Gram Stain Kit (**c** and **d**), which stains Gram-negative bacteria blue and Gram-positive bacteria green. Scale bar represents 20 μm. The images shown are representative of one biofilm formed per each sputa, that was repeated at least two times, yielding the same results.

Interestingly, our system allowed the biofilm formation and growth of different bacterial species simultaneously from the sputum samples. Figure 4 displays representative confocal microscopy images of the biofilms formed from four different sputa (I–IV) (Supplementary Table 3). Different bacterial shapes and morphologies can be seen in the enlarged image of biofilms a) and b) (Fig. 4, sputum I-II), stained with the LIVE/DEAD Viability Kit. Furthermore, the biofilms from sputum III and IV (Fig. 4c, d), stained with the Gram Stain Kit, can be distinguished on the basis of the Gram staining of the different bacteria found. Information concerning the identification and antibiotic susceptibility of the bacteria present in each sputum sample is presented in Supplementary Table 3. Sputum II and IV were found to contain *P. aeruginosa* and *S. aureus* (Supplementary Table 3), which could correspond to the bacilli and staphylococci found in the biofilm (Fig. 4b, d). However, other bacteria also grew, as observed in the confocal images. On the other hand, when the sputa was grown in agar plates at the Microbiology Service at the Vall d’Hebron Barcelona Hospital, only *P. aeruginosa* was identified in the cultures of sputum I and III (Fig. 4a, c, Supplementary Table 3), which can correspond to the bacilli found in the biofilm from sputum I, but no signs of Gram-negative bacilli were found in the biofilm from sputum III, demonstrating the existing variability between the planktonic cells used for microbiological identification and those involved in biofilm growth.

The importance of growing the biofilms directly from sputum samples before analyzing them was corroborated by directly visualizing the sputum by confocal microscopy imaging (Supplementary Fig. 2), showing that it is not possible to directly observe diverse bacteria and their interactions due to the low bacterial concentrations, the presence of eukaryotic cells and the remaining mucus.

### Correlation of confocal microscopy and impedance measurements in the BiofilmChip

To validate the use of the BiofilmChip in antibiofilm drug susceptibility tests, 3-day-old biofilms of *P. aeruginosa* PAO1 (wild-type strain) and *P. aeruginosa* PAET1 (clinically isolated strain) were treated for 24 h with 2 μg/ml and 20 μg/ml ciprofloxacin, respectively. They were imaged with confocal microscopy to calculate the biofilm biomass. As shown in Fig. 3d, the treated biofilm biomass was significantly decreased compared to that of the control biofilm without treatment. Then, to evaluate whether impedance measurements could be correlated to the established confocal microscopy images for biofilm monitoring, a *P. aeruginosa* PAO1 strain encoding a green fluorescent protein (*GFP*) (MK171) was used to grow and easily visualize the biofilm during the time when staining it continuously was not necessary.

**Fig. 5:**
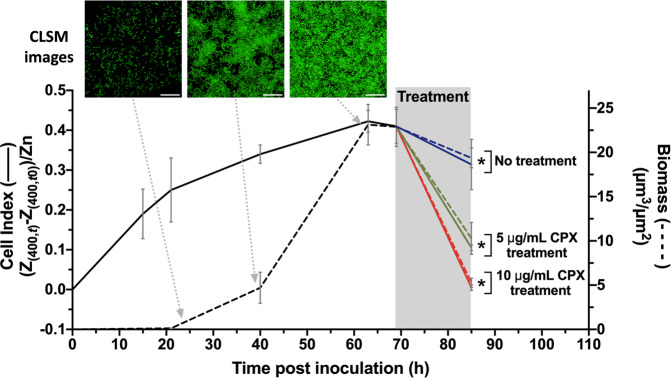
Correlation in real time the biofilm formation and removal using confocal microscopy and impedance measurement. Monitored *P. aeruginosa* MK171 (expressing eGFP) biofilm growth and effect of antibiotic treatment evaluated on the basis of electrical impedance (represented as the CI, left *y*-axis, solid line) and biomass calculated from confocal microscopy images (defined as μm^3^/μm^2^, right *y*-axis, dashed line). In the shaded part, corresponding to the treatment duration, green and red lines represent 5 μg/ml and 10 μg/ml ciprofloxacin treatment, respectively, and the blue line illustrates the control without treatment. The values shown are the average of three independent experiments, and the error bars indicate the standard deviation between the experiments. Representative confocal images from three different time points are shown. Scale bar represents 20 μm. *p < 0.05 in a Pearson’s correlation test.

We next used BiofilmChip devices with integrated interdigitated sensors (see Fig. 1a, b and the Material and Methods). Initially, impedance was measured at frequency values between 40 Hz and 400 kHz every 12 h during the entire biofilm growth period (from before inoculation until after treatment). Measures were plotted in a Bode diagram (shown in Supplementary Fig. 3). Ward et al. proposed different mechanisms on how biofilm growth can interfere in electrode-electrolyte impedance value (i.e., production of redox compounds; biofilm material deposition onto the electrode surface; the presence of microbial cells close to electrode surface; among others)^36^. They suggested that measurements at low frequencies are dominated by charge transfer and mass diffusion of electroactive compounds. In concordance with their results, impedance value at 400 Hz frequency showed substantial differences in impedance at the different steps of the experiment. Accordingly, impedance measurements at 400 Hz and confocal microscopy images were performed at the same time to calculate the cell index (CI) and the biofilm biomass, respectively. CI is a normalized parameter where the impedance value of the blank sample is subtracted from the value measured at a specific time point of the biofilm growth (see the Materials and Methods). In the early stages of biofilm formation, the cells can be imaged under a confocal microscope, but the calculated biomass is nearly zero. The CI, however, varied during the initial biofilm formation stages and thus, can better assess the cell attachment to the substrate as seen in Fig. 5. Thereafter, 69 h old biofilms were treated with ciprofloxacin at 5 μg/ml and 10 μg/ml for 16 h, and the impedance and biomass were measured. Both the CI and biomass presented the same response to the antibiotic at the two concentrations (green and red lines, Fig. 5) or the lack of treatment (blue lines, Fig. 5).

Considering the impedance changes on the basis of antibiotic treatment seen in Fig. 5, the applicability of these impedance measurements to the antibiotic sensitivity of real patient samples was tested (Supplementary Table 3, sputum V–VII). Biofilms were grown from different sputum samples having a pathogen with a known susceptibility profile, and they were treated with different antibiotics. Biofilm impedance diminished in the presence of an effective antimicrobial treatment, as proven by the difference in CI values calculated before and after antibiotic exposure of biofilms formed from sputa V–VII (Supplementary Table 3 and Supplementary Fig. 4). This CI response agrees with the response found from Fig. 5, validating the use of BiofilmChip for biofilm susceptibility testing directly from clinical samples.

## Discussion

Biofilm-related infections are currently a serious threat around the world. The overuse and misuse of antibiotics, along with the increase of biofilm-associated chronic infections is leading to an increasing prevalence of multi-drug resistant bacteria, resulting in a rise in the morbidity and mortality of these infections, which are expected to cause the death of ten million people by 2050^37^. The susceptibility of bacteria embedded in a biofilm to antimicrobial treatment can significantly differ from that of the same free-living bacteria, as it is known that bacteria growing in biofilms have an intrinsically higher antibiotic resistance than their planktonic counterparts due to different mechanisms^38^. Nevertheless, currently, the treatment decisions for biofilm infections are based on susceptibility tests performed in planktonic bacteria immediately after individual bacterial isolation. This makes the treatment choice inappropriate for the effective management of biofilm-associated infections^39^. Moreover, standard antimicrobial susceptibility tests are performed on individual isolates considered relevant by the microbiologist among the full range of growing microorganisms, so in case more than one pathogen is isolated, multiple tests have to be carried out. Taken together, part of the antimicrobial misuse could be attributed to the lack of tools and readily implemented technologies applied in typical microbiology laboratories to easily determine the proper treatment for biofilm infections.

The tool we developed consists of a microfluidic chamber aligned to an interdigitated electrode on which the biofilm grows. It was designed to evaluate biomass formation during the establishment of a biofilm and test antimicrobial drugs and treatments using EIS. Due to the integration of EIS into this BiofilmChip, it is unnecessary to use sophisticated technology such as advanced confocal microscopy, making it suitable for typical microbiology laboratories or industry.

The fabrication cost of the BiofilmChip once you have a specific mask fabrication is cheap (less than one US dollar) and the impedance measurement device can be fabricated easily by an electronic enterprise with cost less than four hundred US dollars. For sure extended manufacturing of all the different BiofilmChip components will reduce the price considerably.

BiofilmChip has several advantages. For example, the microfluidics better mimic growth and resemble a naturally occurring biofilm and reduce the amount of medium and reagents needed to run these continuous biofilm experiments (~16 ml of media per each set of three chambers per day, which is 90% of media reduction compared to the flow cell system). We have optimized this chip’s development by producing different variants of the growth chamber geometry and dimensions to enable optimal biofilm development. It has been documented that the flow cell geometry and the shear stress in the chambers affect biofilm growth^40^. In agreement with that, we observed that both the chamber shape and height were key factors to be considered when reproducing a homogeneous biofilm. It was found that a rectangular shape and 150 μm height were optimal for this purpose, as shown in Fig. 2.

Moreover, an important feature of our device is the inclusion of a prechamber in the design (Fig. 1a), which prevents biofilm disturbances caused by manual inoculations that suddenly increase the flow rate (and consequently the shear stress) inside the chambers. The simulations of the media flow velocity inside the chambers (Supplementary Fig. 1) demonstrated that the design with a prechamber enables a more homogeneous media distribution among the three chambers of the same set, leading to a more uniform biofilm and minimizing the impact of manual injection. Although biofilms in nature are rather heterogeneous^41^, homogeneity is a key factor for further reliable detection of susceptibility under different treatments. As Fig. 3d shows, significant decreases in *P. aeruginosa* PAO1 and PAET1 biofilm biomass were detected after treatment with 2 μg/ml and 20 μg/ml ciprofloxacin, respectively. To our knowledge, the prechamber is an innovative component that has never been published before; therefore, this new component represents a breakthrough in microfluidic devices, where flow rate control is critical.

It is well known that continuous biofilms produced in vitro are more reliable for antibiotic susceptibility studies^42^, as they are more reproducible and similar to the in vivo infection than are in vitro static biofilms performed in microtiter plates. However, the analysis of these continuous biofilms by confocal microscopy is expensive, requires further staining and trained personnel and is extraordinarily time-consuming. We demonstrate an excellent correlation of the biofilm biomass measured via a standardized methodology involving confocal microscopy with the impedance results, which clearly validates the use of EIS to evaluate biofilm growth. Besides, the use of an EIS analyzer is quicker, simpler, and does not require any training. One interesting observation from the use of impedance measurements at 400 Hz is that such measurements can detect the attachment of bacterial cells to the substrate in the early stages of biofilm growth, when usually there aren’t enough cells to be detected as a biofilm by the COMSTAT software analysis of confocal microscopy images (Fig. 5). Once a biofilm is mature, the measures under confocal microscopy are comparable to those under EIS.

Moreover, the impedimetric variations can be used to measure the response of biofilms to antibiotic exposure. In the biofilms in Fig. 5, the CI has the same pattern as the quantified biomass. For the biofilms formed from sputum samples described in Supplementary Table 3 (V–VII), the impedimetric response of the biofilms to the antibiotic was on the same wavelength as the sensitivity reported from the sputum (Supplementary Fig. 4), demonstrating the applicability of the BiofilmChip for antibiofilm testing and choosing the right personalized therapy in the future.

Interestingly, the BiofilmChip was designed to be used for growing biofilms with different characteristics. As far, it has been tested with biofilms composed of different bacterial species (from laboratory use or clinical isolates) and isolated directly from sputum samples obtained from patients suffering Cystic Fibrosis, but the applicability of the BiofilmChip can be extended to different patient samples (sputum from patients suffering from bronchiectasis, urinary infections, etc.) or hypothetically from contaminated surfaces such as medical devices (catheters, cardiac valves, infected joints, etc.) or samples from the food industry (surfaces, etc.), among others.

Under natural conditions, most of these biofilms are formed from a polymicrobial community of bacteria^43^ which interact and affect the other’s pathogenicity and antibiotic susceptibility^44^. Specifically, a clear example is found in chronic infections in the cystic fibrosis lung, which is known as a polymicrobial consortium formed from different bacterial species^45,46^. Under these conditions, the antibiotic concentration needed to remove the formed biofilm is different from the needed to treat isolated planktonic bacteria or even to treat bacteria grown in a monomicrobial biofilm.

Our BiofilmChip enables the growth of polymicrobial biofilms, as it makes the growth of a biofilm directly from a patient sputum sample possible and ensures that no species are excluded, and helps determine the best treatment needed for personalized therapy. For instance, the sputum samples used to form the biofilm shown in Fig. 4b, d) are reportedly from a CF patient suffering from a lung infection by *P. aeruginosa* and *S. aureus* (sputum II and IV in Supplementary Table 3). However, when these sputum samples were directly grown in the BiofilmChip, a diverse population of other microorganisms was found, and the polymicrobial nature of these infections was demonstrated. The combination of confocal microscopy with the impedance measurements in the BiofilmChip would allow to determine which species from the polymicrobial biofilm is affected by the antibiotic, rendering a better treatment, as already described by Müsken et al.^22^, where the evolution of the polymicrobial biofilms in response to different antibiotic treatments was analyzed. However, it has to be taken into account that more species would be able to grow if using different mediums and/or other growing conditions. The antibiotic treatment prescribed for these patients has probably been determined from the individual susceptibility of *P. aeruginosa* and *S. aureus* grown in isolation and planktonically, but as we have previously reported^33^, antibiotic resistance can significantly change in polymicrobial biofilms^47,48^. The importance of individualized diagnostic of biofilm resistance in chronically infected patients was previously highlighted to increase the effectiveness of the treatment^49^. Ongoing work will reveal whether the BiofilmChip allows personalized treatment, ensuring a more accurate diagnosis and a better clinical outcome.

## Methods

### Laboratory and clinically isolated bacterial strains and growth conditions

Wild-type *Pseudomonas aeruginosa* PAO1 strain CECT 4122 (ATCC 15692) and *Staphylococcus aureus* CECT 86 (ATCC 12600) were obtained from the Spanish Type Culture Collection (CECT). The *P. aeruginosa* PAET1 strain isolated from a cystic fibrosis patient suffering from persistant infection and *S. aureus* MRSA were from our laboratory stock^50,51^. *Pseudomonas aeruginosa* PAO1::eGFP (MK171) was kindly provided by Prof. Tim Tolker-Nielsen^52^. To obtain inocula for examination, the strains were cultured overnight at 37 °C in Luria Bertani (LB) liquid medium (Scharlab, Spain) for *P. aeruginosa* and tryptic soy broth (TSB) medium (Scharlab, Spain) for *S. aureus*. Bacterial growth was measured by reading the absorbance at 550 nm (A_550_).

### Obtaining and processing clinical sputum samples

Excess sputum samples (Supplementary Table 3) from cystic fibrosis patients were collected at the Hospital Universitari Vall d’Hebron Microbiology Department. Approval regarding human sample collection and manipulation was obtained from the Clinical Research Ethics Committee (Comitè Ètic d’Investigació Clínica, CEIC) under the number PR(AG)275/2019. Sputum samples were diluted in 10 mM Tris-HCl hypotonic buffer at pH 7.5 (Fisher Scientific, Spain) in a 1:1 ratio and incubated at 4 °C for 5 min before inoculation.

### Microfluidic device design and fabrication: chip fabrication and electrode fabrication

The fabrication of the BiofilmChip (Fig. 1a) involved a multistep procedure using photolithographic and soft lithographic techniques carried out in the MicroFabSpace and Microscopy Characterization Facility, Unit 7 of ICTS “NANBIOSIS” from CIBER-BBN at IBEC. All solvents and chemicals were obtained from Sigma Aldrich unless otherwise specified. The SU-8 photoresist and developer were from MicroChem (Newton, MA). The Ordyl photoresist and developer were from Elga (Italy).

Microfluidic chambers were designed using CAD software (Autodesk, USA), and the master was built over a glass substrate (TED PELLA, Inc., USA) (75 mm × 25 mm). Briefly, the fabrication process started with three consecutive solvent baths (acetone, isopropanol, and ethanol) applied to the glass slide. Following this cleaning protocol, up to three layers of Ordyl SY 550 were laminated on top of the support material using a hot/cold laminator to obtain a smooth attached film surface (150 μm high). Then, the slide was exposed through an acetate photomask to UV light (5 s, 24 mW cm2, 345 nm) in the mask aligner and subsequently placed on a hot plate at 65 °C for 3 min.

The 3D master mold fabrication was finished by developing the Ordyl film using the developer solvent for 3 min. To replicate this mold, a poly(dimethylsiloxane) (PDMS) prepolymer mixture (a curing agent-to-PDMS ratio of 1:10, Sylgard®184, Dow Corning) was placed in a desiccator with a vacuum applied to remove bubbles. The mixture was then poured on top of the Ordyl master to fabricate a PDMS replica and heated overnight at 65 °C. The casted PDMS was peeled off carefully, and inlet and outlet holes were made using a Harris Uni-Core 0.5 mm puncher. A general view of the PDMS BiofilmChip used for biofilm growth and analysis is shown in Fig. 1a. The BiofilmChip includes several sets of independent chambers, each connected to two inlets (one for medium and one for bacterial inoculation), one prechamber, three different growth chambers (C1, C2, C3) and one outlet (Fig. 1b). Each growth chamber was 2 mm wide, 10 mm long and 150 μm high. The BiofilmChip unit was fabricated as three connected chambers (C1, C2 and C3) and considered representative with coherent replicates for straightforward analysis of a given sample.

Gold interdigitated sensors (Fig. 1b) were fabricated onto a glass coverslip to facilitate inspection under a confocal microscope and correlation to impedance measurements. The dimensions and characteristics of the interdigitated electrode were 16 pairs of gold fingers with 75 μm width and 75 μm gap between them. The design of these fingers was selected following the microfluidic chamber size (Fig. 1b). AZ5214 was spread using a spinner to create a uniform layer over the glass substrate. After that, the resin was exposed to UV light in the desired pattern. Then, it was reversed, followed by UV flood light exposure. Next, the glass coverslip was dipped into a solution of AZ Developer 400 K. Then, 20 nm chromium and 80 nm gold were thermally evaporated on glass. After this step, the glass slip was dipped into an AZ Remover 100 solution to remove the photoresist, leaving only the desired pattern’s gold/chromium layer. For bonding to the PDMS microfluidic chamber, an O_2_ plasma cleaner was used to activate both surfaces. PDMS slabs were carefully placed in contact with the glass slide and left on a hot plate to create permanent bonding. The PDMS microfluidic chamber chip was ready to use. Before every assay, the microfluidic device was exposed to UV/ozone for 15 min to make the surface hydrophilic and sterile. Then, the device was filled with sterile Milli-Q water.

### Experimental setup

The BiofilmChip system operated in the three different biofilm formation stages (inoculation, growth and measurement). The final setup is shown in Fig. 1c. LB or TSB +0.2% glucose was stored in a 100 ml bottle connected through Tygon E-3603 tubing (1.5 mm diameter) (DD BioLab) to the microfluidic device. Tubes were attached to the chip by insertion into the inlet and outlet access holes. The medium was pumped through the system with a high-precision Ismatec IPC16 ISM933 multichannel peristaltic pump (Ismatec). Bubble traps (DTU Systems Biology, Technical University of Denmark) were added to the system to avoid the formation of bubbles inside the growth chambers. A constant flow rate of 11.4 μl/min for each set of three chambers was established. The system was filled with media through one inlet, and then, at the inoculation stage, a solution of bacteria (A_550_ ≈ 0.1) or diluted hypotonic buffer sputum sample was pumped into the chip chamber through the other inlet.

At the start of the growth stage, the peristaltic pump was stopped for two hours to allow cell adherence to the glass surface. Then, the medium flowed through the chambers for 65–72 h. The system was previously sterilized with 0.5% v/v sodium hypochlorite in water as previously described^53^, and experiments were carried out at room temperature.

### Confocal laser scanning microscopy and image analysis

*Pseudomonas aeruginosa* and *Staphylococcus aureus* nonfluorescent biofilms were stained with the LIVE/DEAD BacLight Bacterial Viability Kit (Thermo Scientific), consisting of SYTO9 and propidium iodide. Staining of the biofilms formed from sputum was performed with the Bacterial Viability and Gram Stain Kit (Biotium), which differentiates Gram-positive and Gram-negative bacteria. This kit comprises wheat germ agglutinin (WGA) coupled to a CF^TM^-488 fluorophore that stains the walls of Gram-positive bacteria and the dye DAPI, which stains both Gram-negative and Gram-positive bacteria. Biofilms formed with *Pseudomonas aeruginosa* MK171 (expressing eGFP) were visualized under the microscope directly without staining.

All biofilms were visualized under a Zeiss LSM 800 confocal scanning laser microscope (CSLM) using 20X/0.80 air or 63X/1.4 oil objectives. Simulated fluorescence projections and orthogonal sections were generated using ImageJ software, and COMSTAT 2 software was used to quantify the biomass and thickness of the biofilms^54^.

### Biofilm treatment

Biofilms between 65 and 72 h old were treated for 16 or 24 h with different antibiotic concentrations while the medium flow was stopped. After the treatment, the flow was set at 11.4 μl/min for 30 min to wash and exclude detached bacteria affected by the antibiotic from the biofilm.

### Impedance measurements and cell index calculation

We based our monitoring on the cell index (CI) parameter^55^, as we wanted to use an independent, standardized parameter to compare measurements among different samples. The measurements were performed at 400 Hz as we detected bigger changes in bold diagram (see Supplementary Fig. 3).

1 $${{{\mathrm{CI}}}}\left( t \right) = \frac{{Z_{(400,t)} - Z_{(400,t0)}}}{{Z{{{\mathrm{n}}}}}}$$

Where Zn is the corresponding impedance value at 400 Hz of medium with no cells, Z_(400,*t*)_ is the measured impedance at 400 Hz at time point t of biofilm growth and Z_(400,*t*0)_ is the background impedance 2 h after bacteria inoculation, before the biofilm started to grow.

The device used to record EIS values was an Agilent 4294 A 40 Hz–110 MHz precision impedance analyzer. This equipment allowed measuring the impedance along a range of frequencies with an oscilloscope level of 0.5 V. The impedance was measured in the module and phase format and plotted in Bode diagrams. Once the microfluidic device was connected, two impedance measurements were recorded each day, one during the morning and the second one during the afternoon.

### Statistical analysis

Significant differences in biofilm biomass of the ciprofloxacin-treated biofilms vs. non-treated biofilms were calculated using an unpaired *t*-test with the GraphPad Prism 9.0 software. Correlation between Biomass and Cell Index parameters in the biofilm treatment was calculated using Pearson’s correlation with the GraphPad Prism 9.0 software.

### Reporting summary

Further information on research design is available in the Nature Research Reporting Summary linked to this article.

## Supplementary information

Supplementary Information

Reporting Summary

## Data Availability

The authors declare that all relevant data supporting the findings of the study are available within the article and the Supplementary information file. Additional details are also available upon reasonable request.
