# Supplementary material for: A new BiofilmChip device for testing biofilm formation and antibiotic susceptibility
Source: NPJ Biofilms Microbiomes. 2021 Aug 3;7:62. doi: 10.1038/s41522-021-00236-1 (PMC8333102; doi:10.1038/s41522-021-00236-1)

A new BiofilmChip device for testing biofilm formation and antibiotic susceptibility

Núria Blanco-Cabra[1,¶], Maria José López-Martínez[2,3,4,¶], Betsy Verónica Arévalo-Jaimes[1], María Teresa Martin-Gómez[5], Josep Samitier[2,3,4] and Eduard Torrents[1,6,*]

Supplementary Tables and Figures

Supplementary Table 1. Ciprofloxacin Minimal Inhibitory Concentration (MIC) for the *P. aeruginosa* strains used.

***P. aeruginosa* strains**	**Ciprofloxacin MIC (µg/ml)**
**PAO1**	0.25
**PAET1**	2

Supplementary Table 2. Percentage of dead cells (stained in red in the Live/Dead BacLight Bacterial Viability Kit) in the total biofilm biomass of the *P. aeruginosa* PAO1 and PAET1 biofilms treated with ciprofloxacin.

***P. aeruginosa* biofilm Strain**	**Treatment**	**% Dead cell biomass / Total biomass**
**PAO1**	0 µg/ml ciprofloxacin	10.99 % ± 1.35
	2 µg/ml ciprofloxacin	30.17 % ± 1.92
**PAET1**	0 µg/ml ciprofloxacin	15.94 % ± 4.58
	20 µg/ml ciprofloxacin	73.42 % ± 3.51

Supplementary Table 3. Sputum samples from cystic fibrosis patients were used to grow the biofilms, and the cell index was calculated after antibiotic treatment. Information concerning the bacterial species identified and their antibiotic sensitivity was obtained from the Microbiology Service at the Vall d'Hebron Barcelona Hospital Campus.

**Sputum sample**	**Bacterial species identified**	**Antibiotic treatment (sensitivity)**	**Cell Index (CI) change after antibiotic treatment**
I	*P. aeruginosa*	(Not reported)	(Not used)
II	*P. aeruginosa* and *S. aureus*	(Not reported)	(Not used)
III	*P. aeruginosa*	(Not reported)	(Not used)
IV	*P. aeruginosa* and *S. aureus*	(Not reported)	(Not used)
V	*S. aureus*	Ciprofloxacin (Susceptible)<br>Ampicillin (Resistant)	- 0.06<br>+ 0.07
VI	*S. aureus*	Ampicillin (Susceptible)<br>Ciprofloxacin (Resistant)	- 0.93<br>+ 0.72
VII	*P. aeruginosa*	Colistin (Susceptible)<br>Ampicillin (Resistant)	- 0.07<br>+ 0.04

Supplementary Fig. 1. Results of computational fluid dynamics (CFD) simulations to show the effect of a prechamber when loading the sample manually. Figures show contours of velocity (m/s) on the surface of the designed biofilm chip without (a) and with (b) a prechamber.

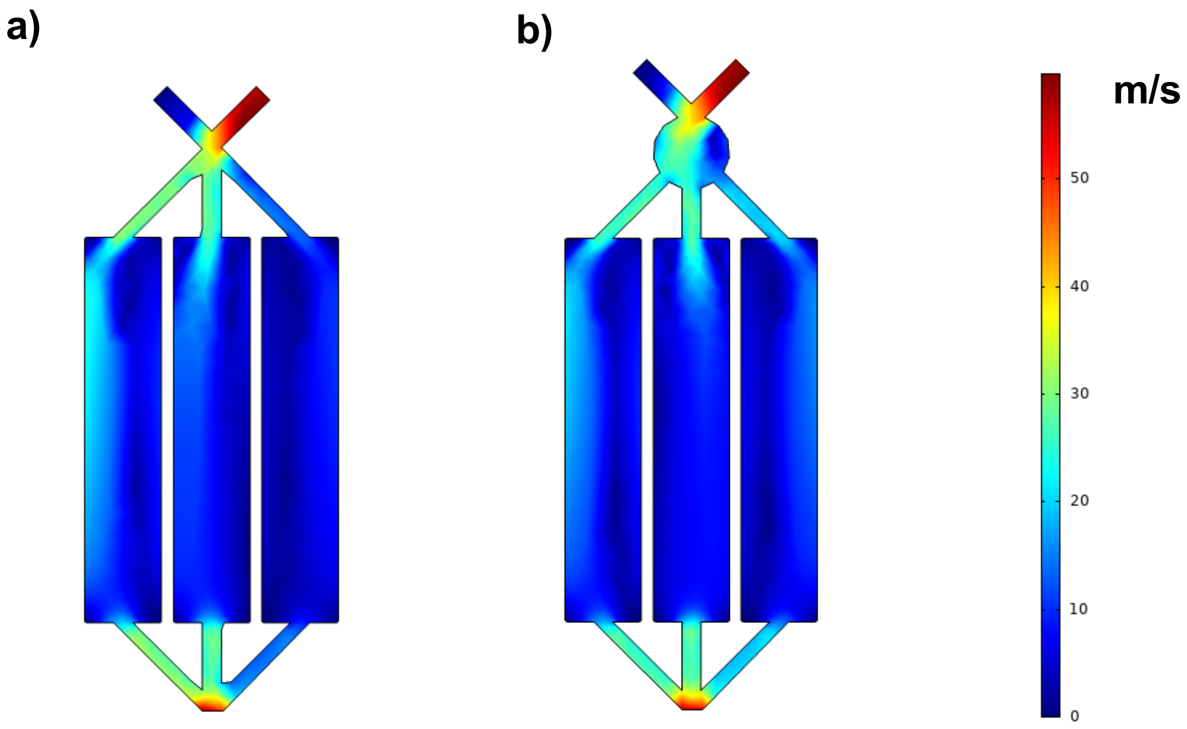

Supplementary Fig. 2. Confocal microscopy images of different sputum samples stained with the Bacterial Viability and Gram Stain Kit. Scale bars represents 10 μm.

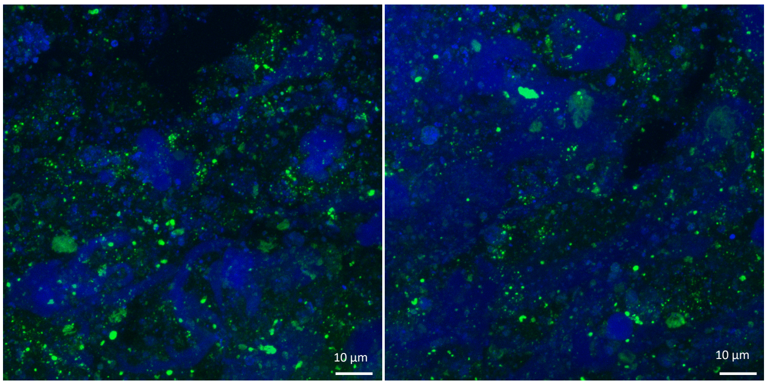

Supplementary Fig. 3. Bode diagram for a treated BiofilmChip chamber. Sweep frequency measurement was between 4 Hz - 400 kHz.

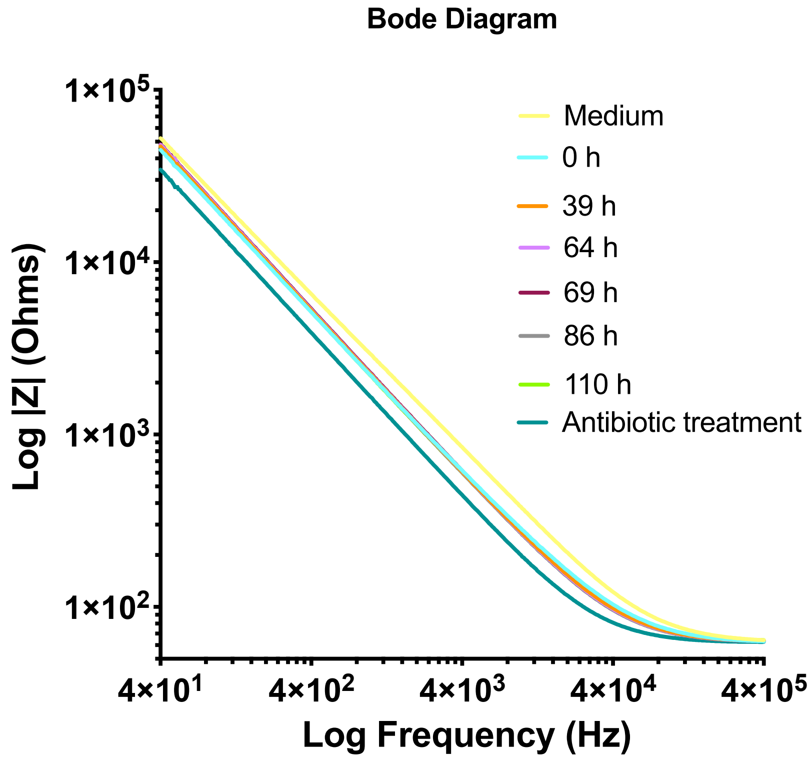

Supplementary Fig. 4. Cell Index change before and after treating the biofilms formed from cystic fibrosis patients' sputa under different antibiotics.

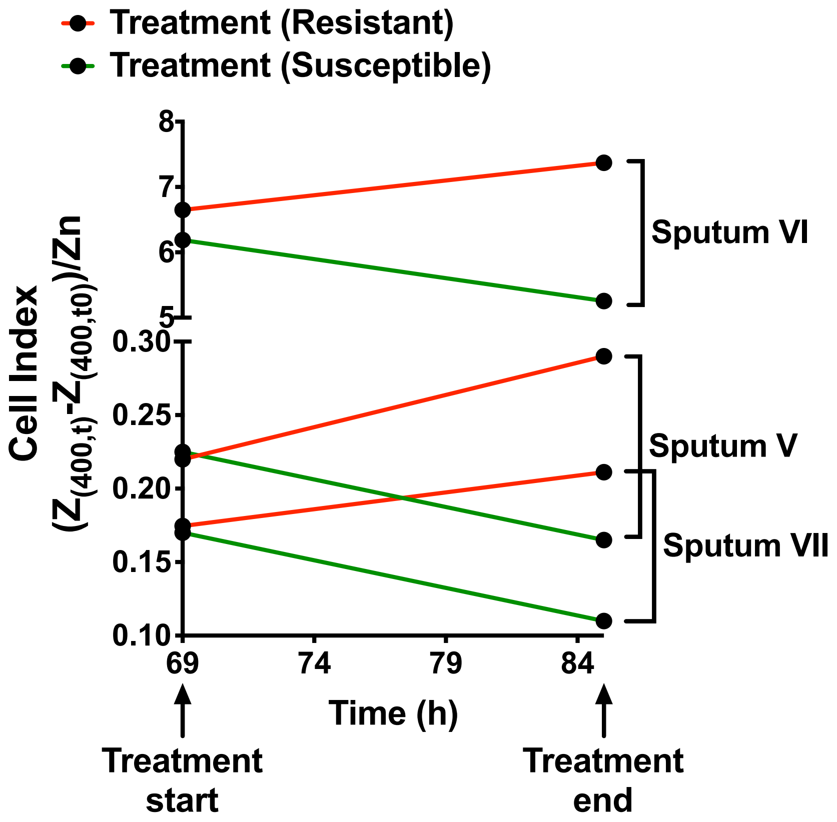

Supplement: Supplementary file 1 — Supplementary Information [file 41522_2021_236_MOESM1_ESM.pdf]
